# Protocol TOP-Study (tacrolimus organ perfusion): a prospective randomized multicenter trial to reduce ischemia reperfusion injury in transplantation of marginal liver grafts with an *ex vivo* tacrolimus perfusion

**DOI:** 10.1186/2047-1440-2-3

**Published:** 2013-03-04

**Authors:** Sebastian Pratschke, Michael Eder, Michael Heise, Silvio Nadalin, Andreas Pascher, Peter Schemmer, Marcus N Scherer, Frank Ulrich, Heiner Wolters, Karl-Walter Jauch, Dirk Wöhling, Martin K Angele

**Affiliations:** 1 Department of Surgery, University of Munich Hospital, Campus Grosshadern, Ludwig-Maximilians-University, Marchioninistrasse 15, 81377 Munich, Germany; 2 Department of Transplantation Surgery, University Medical Center, Johannes Gutenberg University, Langenbeckstrasse 1, 55131 Mainz, Germany; 3 Department of General, Visceral and Transplantation Surgery, University Hospital Tübingen, Eberhard Karls University, Hoppe-Seyler-Strasse 3, 72076 Tübingen, Germany; 4 Department of General, Visceral and Transplantation Surgery, Charité Campus Virchow-Klinikum, Augustenburger Platz 1, 13353 Berlin, Germany; 5 Department of General, Visceral and Transplantation Surgery, Heidelberg University Hospital, Ruprecht-Karls-University, Im Neuenheimer Feld 110, 69120 Heidelberg, Germany; 6 Department of Surgery, University Hospital Regensburg, University of Regensburg, Franz-Josef-Strauss-Allee 11, 93053 Regensburg, Germany; 7 Department of General and Visceral Surgery, Johann Wolfgang Goethe-University Frankfurt am Main, Theodor-Stern-Kai 7, 60590 Frankfurt am Main, Germany; 8 Department of General and Visceral Surgery, University Hospital Münster, Westphalian Wilhelms-University, Waldeyerstrasse 1, 48149 Münster, Germany; 9 DABIO Gesellschaft für Auftragsforschung mbH, Altlaufstrasse 40, 85635 Höhenkirchen, Germany

**Keywords:** Liver transplantation, Organ shortage, Extended donor criteria, Marginal grafts, Tacrolimus, Organ rinse, Graft function, Graft survival

## Abstract

**Background:**

Critical organ shortage results in the utilization of extended donor criteria (EDC) liver grafts. These marginal liver grafts are prone to increased ischemia reperfusion injury (IRI) which may contribute to deteriorated graft function and survival. Experimental data have shown that the calcineurin inhibitor tacrolimus exerts protective effects on hepatic IRI when applied intravenously or directly as a hepatic rinse. Therefore, the aim of the present study is to examine the effects of an *ex vivo* tacrolimus perfusion on IRI in transplantation of EDC liver grafts.

**Methods/Design:**

The TOP-Study (tacrolimus organ perfusion) is a randomized multicenter trial comparing the *ex vivo* tacrolimus perfusion of marginal liver grafts with placebo. We hypothesize that a tacrolimus rinse reduces IRI, potentially improving organ survival following transplantation of EDC livers. The study includes livers with two or more EDC, according to Eurotransplant International Foundation’s definition of EDC livers. Prior to implantation, livers randomized to the treatment group are rinsed with tacrolimus at a concentration of 20 ng/ml in 1000 ml Custodiol solution and in the placebo group with Custodiol alone. The primary endpoint is the maximum serum alanine transamninase (ALT) level within the first 48 hours after surgery; however, the study design also includes a 1-year observation period following transplantation. The TOP-Study is an investigator-initiated trial sponsored by the University of Munich Hospital. Seven other German transplant centers are participating (Berlin, Frankfurt, Heidelberg, Mainz, Münster, Regensburg, Tübingen) and aim to include a total of 86 patients.

**Discussion:**

Tacrolimus organ perfusion represents a promising strategy to reduce hepatic IRI following the transplantation of marginal liver grafts. This treatment may help to improve the function of EDC grafts and therefore safely expand the donor pool in light of critical organ shortage.

**Trial register:**

EudraCT number: 2010-021333-31, ClinicalTrials.gov identifier: NCT01564095

## Introduction

Organ shortage represents a critical problem in transplantation medicine. In 2010, 1192 liver transplantations were performed in Germany as opposed to 1846 new entries on the waiting list (German Organ Transplantation Foundation (Deutsche Stiftung Organtransplantation, DSO), Annual Report, 2010). As a consequence of this discrepancy, there is a noticeable trend towards the utilization of extended donor criteria (EDC) grafts. Data provided by the Eurotransplant International Foundation indicate that the proportion of liver grafts exhibiting one or more EDC increased from 29% in 1997 to 73% in 2010 (Axel Rahmel, Medical Director, Eurotransplant, personal communication). The proportion of grafts with two or more EDC increased from 4% up to 28% over the same time period.

Ischemia reperfusion injury (IRI) is a complex inflammatory, allogen-independent process commonly seen following graft transplantation; however, it is particularly pronounced in marginal organs [1-3], and may contribute to poor graft function and reduced survival in these recipients [4,5]. During ischemia and reperfusion, proinflammatory cytokines such as IL-6 or TNF-α are released into the systemic circulation by Kupffer cells and migrating neutrophils [6]. These molecules induce a complex inflammatory cascade and trigger the generation of reactive oxygen species (ROS) thereby affecting the redox status of the cell [7]. In turn, increased intracellular levels of oxidized glutathione contribute to impairment of the liver’s antioxidative defense system [8]. In addition to sinusoidal congestion caused by endothelial sticking of migrating neutrophils, an imbalance between vasoconstrictive (endothelin-1) [9] and vasodilatatory substances (NO) [10] may directly disturb the hepatic microcirculation. This is considered to be a central pathomechanism for organ dysfunction and primary nonfunction [9,10], especially in marginal liver grafts [11,12]. Besides poor graft quality, the recipients’ health status (model for end-stage liver disease (MELD) score) may also influence the outcome after liver transplantation [13]. The combination of extended criteria donors and poor recipient condition may be responsible for a reduction of graft survival following liver transplantation. In 2011, the 5-year graft survival rate in Germany was 52.6% compared to the international mean of 66.2% (data provided by DSO, Collaborative Transplant Study (CTS)).

Therefore, strategies must be developed to improve the function and survival of EDC organs. Several experimental models have shown that tacrolimus preconditioning before liver transplantation has protective effects (Table 1). The authors have recently demonstrated that an *ex vivo* tacrolimus flush reduces IRI in a model of experimental liver transplantation in rats [14]. Based on these experimental findings, a study protocol of a single *ex vivo* tacrolimus rinse prior to reperfusion in marginal livers was developed (Trial register: EudraCT number: 2010-021333-31, ClinicalTrials.gov identifier: NCT 01564095). The aim of the TOP-Study (tacrolimus organ perfusion) is to reduce hepatic IRI and improve long-term organ survival following transplantation of marginal livers.

**Table 1:** Tacrolimus and ischemia reperfusion injury experimental animal studies

**Author**	**Cold vs warm ischemia**	**Ischemic time**	**Species**	**TAC-application:**	**End points**	***P***
				**Systemic vs organ rinse**		
Sakr *et al*., 1991 [15]	Warm	45 minutes	Rat	Systemic	Survival, aminotransferases, LDH	<0.05
Kawano *et al*., 1995 [16]	Warm	60 minutes	Rat	Systemic	Microcirculation, aminotransferases	<0.05
Kawano *et al*., 1996 [17]	Cold	80 minutes	Rat	Systemic	Lipid peroxidation, aminotransferases	<0.05
Garcia-Criado *et al*., 1997 [18]	Warm	90 minutes	Rat	Systemic	Survival	<0.01
					ROS, cytokines, aminotransferases, neutrophil infiltration	<0.05
Takeichi *et al*., 2009 [19]	Warm	50 minutes	Rat	Systemic	Aminotransferases, neutrophil activation	<0.05
Huser *et al*., 2009 [20]	Cold	120 minutes	Rat	Systemic	Aminotransferases, histology	0.001
Pratschke *et al*., 2012 [14]	Cold	24 hours	Rat	Organ rinse	Aminotransferases, glutathione metabolism	<0.05

LDH, lactate dehydrogenase; ROS, reactive oxygen species; TAC, tacrolimus.

## Hypothesis and endpoints

The hypothesis of the study is that a single *ex vivo* tacrolimus perfusion prior to reperfusion reduces IRI and improves long-term graft survival. The primary endpoint is the maximum alanine transaminase (ALT) level within the first 48 hours following liver transplantation. Secondary endpoints are ALT and aspartate transaminase (AST) levels, graft function (prothrombin time, bilirubin), and creatinine on days 1, 2, 4 and 7. In addition, the study documents graft and patient survival, histologically confirmed rejection, as well as ischemic-type biliary lesions (ITBL).

## Methods

The TOP-Study is an investigator-initiated, prospective, randomized trial comparing the *ex vivo* perfusion of marginal livers with tacrolimus to placebo prior to transplantation. The main inclusion criterion is the presence of two or more EDC. IRI is assessed by serum ALT and AST levels over a period of 7 days. Following this period, organ and patient survival, bile duct complications, rejections and organ function are monitored for 1 year. The TOP-Study is sponsored by the University of Munich Hospital with financial support provided by a grant from Astellas Pharma GmbH, München, Germany. Research and organizational support is provided by the contract research organisation (CRO) DABIO Gesellschaft für Auftragsforschung mbH, Höhenkirchen, Germany.

### Inclusion and exclusion criteria

The study includes patients undergoing liver transplantation in the participating centers who meet the following criteria: chronic terminal liver failure, over 18 years of age, first liver transplantation, and informed, signed consent by the recipient. Donor organs exhibiting two or more EDC according to the Eurotransplant Manual for extended criteria liver donors (Table 2) are included [21].

**Table 2:** Extended donor criteria (EDC)

**Criteria**	
Donor age	>65 years
Macrovesicular steatosis	>40% (macroscopy or biopsy)
BMI	>30
Sodium	>165 mmol/l
ICU stay and ventilation	>7 days
Cold ischemia time	>13 hours
AST	>99 u/l
ALT	>105 u/l
Bilirubin	>3 mg/dl (>51 μmol/l)
Application of epinephrine	

Criteria according to the Eurotransplant Manual for EDC in liver grafts [21]. BMI, body mass index; EDC, extended donor criteria; ICU, intensive care unit; ALT, alanine transaminase; AST, aspartate transamianse.

Patients receiving split liver and multiorgan transplantations are excluded as well as those undergoing retransplantation, high urgency transplantation or pediatric transplantation. In addition, recipients with extrahepatic malignant diseases, and organs from donors with hepatitis B or C infection, are excluded.

### Perfusion procedure

**Figure 1:**
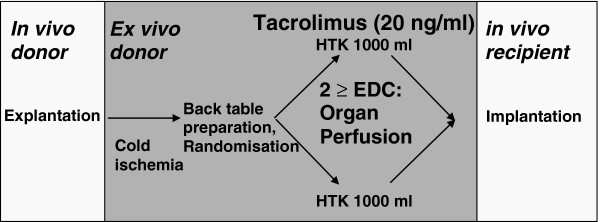
**Flow chart of tacrolimus organ perfusion.** EDC, extended donor criteria; HTK, histidine-tryptophan-ketoglutarate.

Livers are perfused with 1000 ml of the rinse solution from a height of 100 cm without additional pressure using polyvinylchloride (PVC)-free infusion systems with a 12 gauge cannula. The portal vein and the common hepatic artery are flushed sequentially, with 500 ml each. In the test arm, tacrolimus is added to 1000 ml Custodiol histidine-tryptophan-ketoglutarate (HTK) solution at a concentration of 20 ng/ml. A total of 20 μg tacrolimus is applied. In the placebo group livers are perfused with 1000 ml Custodiol. The perfusion procedure is performed at the end of the back-table preparation at least 1 hour before reperfusion. The duration of the perfusion does not exceed 15 minutes (Figure 1). Prior to reperfusion livers are flushed *in situ* with 500 ml of the recipients’ blood.

### Liver transplantation and postoperative immunosuppressant regimen

Liver transplantation is performed according to the standard clinical practice at each center. Immunosuppression during the first 7 postoperative days is tacrolimus-based. Thereafter, a tacrolimus-based immunosuppressive regimen is suggested but not mandatory. Additional immunosuppression, that is, corticoids, is administered at the discretion of the treating clinician.

### Follow-up

**Figure 2:**
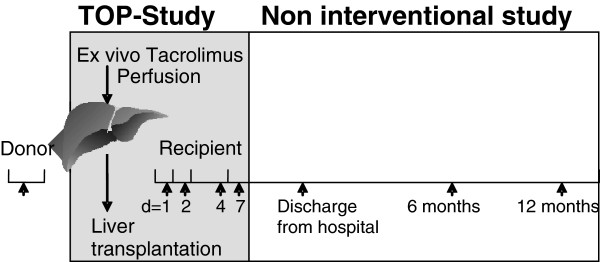
Study visits TOP-Study (tacrolimus organ perfusion).

The present trial includes a 7-day interventional study regulated by the German Pharmaceuticals Act (Arzneimittelgesetz, AMG) and a non-interventional study (NIS) over 1 year (Figure 2). During the entire study period, the monitoring of safety and data is performed according to Good Clinical Practice (GCP) guidelines. Data management and CRO duties are performed by the DABIO Gesellschaft für Auftragsforschung mbH, Höhenkirchen, Germany.

### Study visits

To characterize IRI and graft function serum ALT/AST, prothrombin ratio and bilirubin are measured on postoperative days 1, 2, 4, 7, as well as 6 and 12 months following liver transplantation (Figure 2). Moreover, graft and patient survival, bile duct complications and histologically confirmed rejections are assessed.

### Sample size, statistical analysis, randomization

The primary endpoint of the study is the maximum serum ALT level within 48 hours following liver transplantation, which reflects the degree of acute hepatocellular injury. Non-parametric analysis using Wilcoxon rank-sum test is performed to compare the maximum ALT levels in grafts treated with tacrolimus versus placebo. Based on a previous study using non-marginal healthy grafts, an effect size of approximately 0.5 was calculated [22]. In experimental studies, therapies for the treatment of IRI were more effective in steatotic livers [23,24]. Since recipients of marginal organs are incorporated in the present study, the predicted improvement in postoperative ALT levels should be higher than in non-marginal grafts. Thus, an effect size of 0.7 was considered appropriate for the sample size calculation. The power of the test is 80% at a significance level of 0.05. Therefore, sample size estimation (nQuery Advisor 6.1, Statistical Solutions, Saugus, MA, USA) for two unpaired samples using the Wilcoxon rank-sum test with an expected dropout rate of 15% results in an estimated sample size of 86 (43 tacrolimus vs 43 placebo).

To homogenize the patient collective only marginal organs with two or more EDC are included. Nonetheless, all EDC may affect the primary endpoint. Since documentation of EDC is required for patient inclusion, those parameters will be analyzed as potential confounders. Moreover, recipient age will also be registered.

### Participating centers

The Departments of Surgery of the following German university hospitals are participating in this trial: Charité Campus Virchow-Klinikum, Berlin; Johann Wolfgang Goethe-University, Frankfurt am Main; Johannes Gutenberg University, Mainz; Westphalian Wilhelms-University, Münster; Ruprecht-Karls-University, Heidelberg; University of Regensburg; Eberhard Karls University, Tübingen; and Campus Grosshadern, Ludwig-Maximilians-University, Munich.

### Ethics and safety

Protocol version 2.1 has been approved by the local ethic committees of the ethics committee of the university of Munich. The study complies with the Declaration of Helsinki and GCP guidelines. Informed consent is obtained from each patient in written form prior to randomization. The patient is informed about the nature, duration and possible consequences of the trial by an investigator specifically registered for this trial.

### Current status (October 2012)

Study permission by the Federal Institute for Drugs and Medical Devices (Bundesinstitut für Arzneimittel und Medizinprodukte, BfArM) was received on 29 July 2011 and ethics committee approval on 23 August 2011. Version 2.1 of the protocol is active. To date (October 2012), seven centers (Berlin, Frankfurt, Heidelberg, Mainz, Munich, Regensburg and Tübingen) have been initiated and 17 patients have been recruited for the study. Estimated closure for recruitment for the study will be 31 December 2013. One year thereafter the study will be closed. Data calculation will require 6 months. A finalized report of the study is expected for July 2015.

## Discussion

Organ shortage and the consecutive transplantation of EDC grafts remain an unsolved problem in organ transplantation. Marginal organs are increasingly accepted, which is associated with increased acute IRI [4,23] and diminished graft survival [5,25,26]. An increased susceptibility of marginal organs to the pathomechanisms of IRI is discussed as a potential cause for the impaired outcome of these grafts [2,3]. Thus, clinically relevant strategies must be developed to prevent IRI in marginal organs.

Several experimental studies have demonstrated protective effects of tacrolimus on IRI following liver transplantation [14,17,20]. Despite their promising results, these models were based on systemic donor preconditioning, which is logistically difficult to incorporate into clinical practice due to the existing organ allocation practice in the Eurotransplant zone.

An *ex vivo* tacrolimus treatment may represent a solution to this problem. Recent experimental data indicates a protective effect of an *ex vivo* tacrolimus rinse in a model of experimental liver transplantation in rats [14]. Preservation of intracellular glutathione levels was suggested as a potential mechanism in this study. The calcineurin inhibitor tacrolimus acts through a blockade of the intracellular calcineurin-calmodulin complex. This blockade inhibits the calcium-dependent phosphorylation of the nuclear factor of activated T cells (NFAT). As a consequence, IL-2, which is normally involved in the activation of CD4+ and CD8+ T cells, and the IL-2 receptor are downregulated. Thus, the inactivation of T cells is regarded as the central mechanism in the immunosuppressant properties of tacrolimus [27,28].

In addition, tacrolimus might attenuate allogen-independent hepatic IRI, which is characterized by the release of a complex cascade of cytokines including IL-6 and TNF-α, the generation of ROS, the accumulation and transmigration of different cell types (that is, lymphocytes, neutrophils, platelets), as well as alterations of the microcirculation potentially causing graft dysfunction or even non-function [6]. In this respect, T cells have been shown to be critically involved in the induction of IRI of the liver [29-32]. A rapid recruitment of CD4+ T cells in hepatic sinusoids as early as 30 minutes after reperfusion is followed by their migration through the endothelial barrier to injured hepatic tissue [30]. Although CD4+ T cells themselves are not cytotoxic, they release a panel of cytokines, chemokines and adhesion molecules which are potentially harmful to the organ. Moreover, CD4+ T cells interact with platelets and Kupffer cells which further aggravate IRI [33]. However, it has yet to be determined whether tacrolimus affects IRI after liver transplantation via CD4+ T cells.

Neutrophils are also actively involved in hepatic IRI. The accumulation of neutrophils congests hepatic sinusoids and leads to the release of proinflammatory cytokines (that is, TNF-α and IL-6), as well as ROS [34]. Adhesion molecules such as P-selectin and ICAM-1 are involved in the process of neutrophil recruitment [35]. The application of tacrolimus decreases the expression of these adhesion molecules, thereby attenuating neutrophil recruitment [36,37]. In addition, direct suppressive effects of tacrolimus on the activation of Kupffer cells, which also release proinflammatory cytokines have been demonstrated *in vitro*[38]. This anti-inflammatory effect of tacrolimus was also evident in human liver biopsies after the transplantation of organs rinsed with tacrolimus [39].

With respect to the microcirculation, direct effects of tacrolimus on the expression of vasoconstrictive substances (endothelin-1) in endothelial cells have been shown, which might further improve hepatic microcirculation [40]. Increased levels of ROS are known to be involved in the pathogenesis of IRI. The application of tacrolimus *in vivo* is associated with a reduction of ROS [18]. Recently, a rat model of liver transplantation demonstrated that tacrolimus increases glutathione metabolism, which in turn may protect organ function by reducing ROS toxicity [14]. Tacrolimus has also been found to exert anti-apoptotic effects by preventing Fas-induced apoptosis in human hepatocytes *in vitro*[41], as well as in an *in vivo* model of IRI in rats [42]. A decrease in liver apoptosis may contribute to persisting protection of cellular integrity. In summary, several potentially synergistic mechanisms for the protective effects of tacrolimus in the setting of ischemia-reperfusion injury have been proposed.

Preliminary clinical data have shown beneficial results of tacrolimus preconditioning in human liver transplantation (Table 3). In addition, the tacrolimus rinse procedure has been tested clinically in a phase I trial (Table 3). In a previous trial, Peter *et al*. demonstrated a significant reduction of aminotransferase levels following the transplantation of normal livers rinsed with 20 ng/ml tacrolimus [22]. Although the results of this trial were promising, the clinical impact was limited by the small number of patients included (n = 20). In a similar clinical study, Kristo *et al*. recently failed to show a reduction in ALT levels on day 6 after transplantation [39]. However, the study population was relatively small, and, as most patients received healthy organs, the results cannot be directly compared to a study of marginal grafts. Postoperative aminotransferase levels in the Kristo *et al*. study were generally quite low, with serum ALT levels in the control group reaching almost normal levels 6 days after transplantation [39]. Nevertheless, the authors showed an impressive reduction in precursors of proinflammatory enzymes following tacrolimus rinse [39].

**Table 3:** Clinical studies of tacrolimus rinse in liver transplantation

**Author**	**Number of patients**	**Result**		***P***
		**Tacrolimus**	**Placebo**	
St Peter *et al*., 2003 [21]	20	AST (IU/l)	AST (IU/l)	0.02
		day 1: 604	day 1: 1294	
		day 2: 683	day 2: 934	
Kristo *et al*., 2011 [38]	26	ALT (IU/l)	ALT (IU/l)	0.88
		day 6: 79	day 6: 101	

ALT, alanine transaminase; AST, aspartate transaminase.

In the TOP-Study, livers are treated with a single *ex vivo* tacrolimus rinse prior to implantation, with the aim of reducing graft damage and secondarily improving the long-term course of EDC grafts. The maximum ALT level within the first 48 hours following liver transplantation was chosen as a clinical marker of hepatic injury and used to estimate the degree of IRI. Aminotransferases have been shown to be an appropriate marker of hepatic IRI in a number of studies. Puhl *et al*. demonstrated an inverse correlation between microcirculation, a key factor in the development of IRI, and serum ALT/AST levels in human liver transplantation [43]. Moreover, EDC organs, which are associated with increased levels of IRI, display significantly elevated ALT/AST levels [4]. In addition to assessing acute IRI, the TOP-Study assesses graft survival during a 1-year follow-up period. Although the impact of acute graft injury on long-term survival is discussed controversially in the literature, there is strong evidence that IRI correlates significantly with long-term graft survival [44].

The tacrolimus concentration of 20 ng/ml was chosen in the present trial based on safety data from previous studies [22,39]. At this dosage no adverse effects related to the tacrolimus treatment have been reported. The 20 μg of tacrolimus dissolved in 1000 ml of Custodiol to form the rinse solution represents a minute fraction of the 1.75 × 10^3^ μg per day of tacrolimus administered intravenously to a 70 kg adult. If even 80% of the tacrolimus in the rinse solution reached the systemic circulation, the drug level would be below the detection limit of 3 ng/ml. Therefore, the rinse solution seems to have local effects in the liver graft, rather than contributing to systemic immunosuppression.

In summary, a tacrolimus rinse could represent a new strategy to reduce IRI and improve organ survival in EDC organs in liver transplantation. A reduction of organ damage in marginal grafts may allow the acceptance of more EDC organs, even in patients with high MELD scores, thereby safely expanding the donor pool in liver transplantation.

## Abbreviations

ALT: alanine transaminase; AMG: German Pharmaceuticals Act (Arzneimittelgesetz); AST: aspartate transaminase; BfArM: Federal Institute for Drugs and Medical Devices (Bundesinstitut für Arzneimittel und Medizinprodukte); CRO: contract research organization; CTS: Collaborative Transplant Study; DSO: German Organ Transplantation Foundation (Deutsche Stiftung Organtransplantation); EDC: extended donor criteria; GCP: Good Clinical Practice; HTK: histidine-tryptophan-ketoglutarate; IL: interleukin; IRI: ischemia reperfusion injury; ITBL: ischemic-type biliary lesions; LDH: lactate dehydrogenase; MELD: model for end-stage liver disease; NFAT: nuclear factor of activated T cells; NIS: non-interventional study; NO: nitric oxide; PVC: polyvinylchloride; ROS: reactive oxygen species; TAC: tacrolimus; TNF: tumor necrosis factor; TOP: tacrolimus organ perfusion.

## Competing interests

The study is financed by a grant from Astellas Pharma GmbH, München, Germany.

## Authors’ contributions

SP performed experimental work, participated in the design of the study and wrote the manuscript. ME participated in the coordination of the study and helped to draft the manuscript. MH, SN, AP, PS, MS, FU and HW participated in performing the study (liver transplantation, organ rinse) and helped to draft the manuscript. KWJ participated in the study design and helped to draft the manuscript. DW participated in the study design, in coordination of the study and in statistical analysis. MA conceived the design of the study and helped to draft the manuscript. All authors read and approved the final manuscript.
